# Development of a water-dispersible antimicrobial lipid mixture to inhibit African swine fever virus and other enveloped viruses

**DOI:** 10.1016/j.virusres.2024.199516

**Published:** 2024-12-25

**Authors:** Joshua A. Jackman, Roza Izmailyan, Rafayela Grigoryan, Tun Naw Sut, Abel Taye, Hovakim Zakaryan, Charles C. Elrod

**Affiliations:** a School of Chemical Engineering and Translational Nanobioscience Research Center, Sungkyunkwan University, Suwon 16419, Republic of Korea; b Institute of Molecular Biology of NAS, Hasratyan 7, Yerevan 0014, Armenia; c Natural Biologics Inc., Newfield, NY 14867, USA

**Keywords:** Enveloped virus, African swine fever virus, Influenza A virus, Antiviral, Monoglycerides, Lactylates

## Abstract

Medium-chain antimicrobial lipids are promising antiviral agents to inhibit membrane-enveloped viruses such as African swine fever virus (ASFV) and influenza A virus (IAV) in livestock applications. However, current uses are limited to feed pathogen mitigation due to low aqueous solubility and the development of water-dispersible lipid formulations is needed for broader application usage. In this study, we report a water-dispersible antimicrobial lipid mixture of monoglycerides and lactylates that can inhibit ASFV and IAV and exhibits antiviral properties in drinking water and feed matrices. The lipid mixture reduced the viral infectivity of membrane-enveloped ASFV and IAV in aqueous solution in a dose-dependent manner but was inactive against non-enveloped encephalomyocarditis virus (EMCV). Additional ASFV experiments supported that the lipid mixture is virucidal, which was corroborated by polymerase chain reaction (PCR) experiments. Feed mitigation experiments demonstrated that the lipid mixture can also inhibit ASFV infectivity and affected the conformational properties of ASFV p72 structural protein in virus-spiked feed. Mechanistic experiments identified that the lipid mixture rapidly disrupted phospholipid membranes in a micelle-dependent manner, which aligns with the virological data while higher concentrations were needed for virucidal activity than for the onset of membrane disruption. These findings support that water-dispersible antimicrobial lipid mixtures can effectively inhibit ASFV and IAV and have practical advantages for drinking water applications compared to existing medium-chain antimicrobial lipid mitigant options that are formulated as dry powders or oils for in-feed applications.

## Highlights

• A water-dispersible antimicrobial lipid mixture with virucidal properties was developed.
• Lipid mixture inhibited enveloped ASFV and IAV and was inactive against non-enveloped EMCV.
• Lipid mixture inhibited ASFV infectivity and reduced antigen levels in spiked feed.
• Antiviral properties of mixture were related to micelle-dependent membrane disruption.

## 1 Introduction

The development of antiviral mitigants that can be incorporated into drinking water and feed matrices is a high priority for livestock disease prevention and treatment (Andres and Davies, 2015; Wales et al., 2010). Among known viral pathogen risks, the highly lethal African swine fever virus (ASFV) affecting swine populations is a key biosecurity challenge that lacks effective vaccines or therapeutics and anti-ASFV mitigants are needed urgently (Alarcón et al., 2021; Niederwerder, 2021; Shurson et al., 2023; Urbano and Ferreira, 2022). In addition to ASFV, other persistent viral threats include porcine reproductive and respiratory syndrome virus (PRRSV) and porcine epidemic diarrhea virus (PEDV) in swine populations as well as influenza A virus (IAV) in livestock populations (Salvesen and Whitelaw, 2021; Stewart et al., 2020). The reduced usage of formaldehyde as a general-purpose antiviral mitigant due to regulatory restrictions in some jurisdictions further heightens the need to develop new classes of regulatory compatible antiviral mitigants (Gosling et al., 2021; Pelyuntha et al., 2022).

Naturally occurring antimicrobial lipids such as medium-chain fatty acids (MCFAs) with 6–12 carbon long, saturated hydrocarbon tails are a promising class of antiviral mitigants that have favorable regulatory profiles (*e.g.*, widely approved for safe use in animal feed and human food products) and can inhibit membrane-enveloped viral pathogens such as ASFV in liquid and feed (Jackman et al., 2020a; Niederwerder et al., 2021). MCFAs and related mitigants such as medium-chain monoglycerides and lactylates exhibit antiviral activity by disrupting the outer phospholipid bilayer that surrounds enveloped virus particles (Gooran et al., 2023; Thormar et al., 1987). Notably, it has been shown that medium-chain monoglycerides like 12-carbon long glycerol monolaurate (GML) can inhibit ASFV and other enveloped swine viruses even more potently than MCFAs (Jackman et al., 2020b; Phillips et al., 2022). Within the mitigation scope, medium-chain antimicrobial lipids have emerged as a promising substitute to replace formaldehyde for feed decontamination purposes while some mitigants in this class have also recently demonstrated potential effectiveness for *in vivo* disease prevention and treatment in livestock animals. From a translational perspective, usage in feed matrices has proven particularly effective because, in principle, the mitigant can be applied in various possible phases (*e.g.*, solid powder, spray dispersion, oil) and directly mixed into the feed.

However, the practical use of medium-chain antimicrobial lipid mitigants like MCFAs and GML in other matrices such as drinking water has been tempered by typical solid phase characteristics at ambient temperatures and low aqueous solubility, which arises from the hydrophobic chain of the lipid molecules (Prajapati et al., 2011). In general, a longer chain increases antiviral potency to work at lower mitigant concentrations but there is a tradeoff between solubility and potency (Wang and Johnson, 1992). To partially address this issue, a few studies have described the use of organic solvents and/or synthetic polymer additives for mitigant solubilization but these approaches are not translatable to industry use (Fletcher et al., 2020; Hierholzer and Kabara, 1982; Niederwerder et al., 2021; Zhang et al., 2016). There remains an outstanding need to develop water-miscible antimicrobial lipid mixtures that can be prepared at high stock concentrations and are readily dispersible in aqueous suspensions. In principle, such mixtures could be directly added to drinking water lines while also still being useful for mitigating ASFV in feed, which is a reported transmission vector (Niederwerder et al., 2019).

The objective of the present study was to develop a water-dispersible antimicrobial lipid mixture that contains a high fraction of medium-chain monoglycerides and lactylates, including GML, in order to inhibit enveloped viruses such as ASFV and IAV in aqueous liquid and feed matrices. Viral infectivity experiments with enveloped ASFV and IAV and non-enveloped encephalomyocarditis virus (EMCV) demonstrated that the lipid mixture selectively inhibited enveloped viruses, which was verified by membrane disruption experiments. The liquid-phase antimicrobial lipid mixture was also effective at mitigating ASFV in feed and antiviral concentrations of the mixture were nontoxic according to cell viability measurements.

## 2 Materials and methods

### 2.1 Antimicrobial lipid formulation

A laboratory-scale prototype formulation that contained various antimicrobial lipids was provided by Natural Biologics Inc. (New York, USA). The carrier-free antimicrobial lipid mixture is in the liquid phase at room temperature and includes ∼40 % C_3_ monoglyceride (glycerol monopropionate), ∼10 % C_12_ monoglyceride (glycerol monolaurate), and ∼8 % C_8_, ∼8 % C_10_, ∼12 % C_12_ lactylates along with glycerin and sodium. The mixture components were selected based on combining medium-chain antimicrobial lipids with other molecules that can aid solubilization. The resulting mixture was directly dispersible in aqueous liquid conditions such as phosphate-buffered saline (PBS) and cell culture media. Volumetric dilutions (v/v) were used for solution-phase assays and are reported in X:Y units whereby X was the relative volume of the lipid mixture that was added to the appropriate solvent and Y is the relative total volume of the lipid mixture and solvent together. For feed experiments, the lipid mixture was applied based on the inclusion rate (wt%), whereby a defined amount of the lipid mixture was added to the feed based on the corresponding density of the lipid mixture (∼1.251 g/cm^3^ at 25 °C).

### 2.2 Virus and cell culture

The ASFV BA71V strain and EMCV Columbia-SK strain were grown in commercially supplied Vero cells of African green monkey kidney origin while the IAV A/WSN/33 strain was grown in commercially supplied MDCK cells of canine kidney origin. Virus titration was performed by a cytopathic effect (CPE) assay in a 10-fold serial dilution format, as previously described (Jackman et al., 2020b). Viral titer quantification was determined by the Spearman-Kärber endpoint method and reported in terms of log 50 % tissue culture infective dose (TCID_50_) per mL (log TCID_50_/mL) units (Lei et al., 2021). Both cell lines were maintained and propagated at 37  °C in Eagle's minimum essential medium (EMEM) (Lonza, Belgium) that was supplemented with 10 % fetal bovine serum (FBS), 2 mmol/L *L*-glutamine, 100 international-units/mL penicillin, and 100 μg/mL streptomycin (Sigma-Aldrich, Germany).

### 2.3 Cell viability

Cell viability was investigated by the MTT assay, as previously described (Arabyan et al., 2021). Cells in a 96-well cell culture plate (2 × 10^4^ cells per well) were treated with different lipid mixture doses (1:2 to 1:200 dilutions in cell culture medium). Treated cells were incubated for 24 h at 37 °C in a 5 % CO_2_ environment. The medium was then removed and MTT solution was added, followed by incubation at 37 °C for 3 h After sample processing, colorimetric measurements were performed on a microplate reader at 570 nm and relative cell viability was determined.

### 2.4 Virucidal assay in solution

A virus suspension containing 1.6  ×  10^5^ TCID_50_ ASFV, 1  ×  10^5^ TCID_50_ IAV, or 1  ×  10^5^ TCID_50_ EMCV particles per well was incubated with different dilutions of the lipid mixture for 1 h at room temperature. Then, the treated virus suspension was diluted 20-fold before adding the diluted suspension to infect 2  ×  10^4^ Vero or MCDK cells in a 96-well cell culture plate. After 1-h incubation at 37  °C, the cells were extensively washed with PBS and then EMEM supplemented with 3 % FBS was added. Cell infection was allowed to proceed until complete CPE was developed in virus-only control wells (usually 3–4 d post-infection). Then, the cell supernatants were collected and viral titers in the supernatants were determined by a CPE-based titration assay. Serial dilutions of the supernatants were prepared and inoculated onto Vero cells in a 96-well cell culture plate (6 wells per dilution). The number of wells that were infected was then determined for each virus dilution, and the viral titer was calculated by the Spearman-Kärber method. For ASFV, similar experiments were also performed using the real-time polymerase chain reaction (PCR) method, as previously described (Jackman et al., 2020b). Primers for the ASFV p72 gene were used and the data are reported in terms of cycle threshold (Ct) units.

### 2.5 Antiviral assay in solution

An ASFV suspension (multiplicity of infection: 0.2 TCID_50_ per cell) was incubated with different dilutions of the lipid mixture (1:10, 1:25, 1:50, 1:100, 1:200 plus virus-only control) for 1 h at room temperature, as previously described (Jackman et al., 2020b). The treated virus samples were then added to Vero cells seeded at 2  ×  10^5^ cells per well in a 24-well culture plate. Cell infection was allowed to proceed until complete CPE was developed in virus-only control wells (around 3–4 d post-infection). Afterwards, viral titers in the supernatants were quantified by a CPE-based titration assay as described above.

### 2.6 Anti-ASFV feed mitigation assay

Two grams of commercial swine feed (Aktifeed Grow 20 %, Mustang Nutrition Technology, Moscow, Russia) were mixed with the lipid mixture by vortexing at the intended inclusion rate. Then, each feed sample was incubated with 100 μL of EMEM containing ASFV at 10^6^ TCID_50_ dosage for 30 min or 24 h at room temperature. After incubation, 20 mL of fresh EMEM was added to each feed sample and centrifuged at 3600 × *g* for 40 min (4  °C). The supernatant was collected and aliquots were analyzed in virus recovery and ELISA tests. For viral recovery analysis, 90 % confluent Vero cells in a 96-well culture plate were inoculated with the supernatant diluted in a 10-fold series (6 wells per dilution). After 96 h, viral titers were determined by CPE-based assay. For ELISA analysis, the level of conformationally intact ASFv p72 protein was measured by using a commercially available ELISA kit (INgezim PPA DAS, 3465RD, Ingenasa, Madrid, Spain) and a colorimetric reader, as previously described (Jackman et al., 2020b). The optical density (OD) of each sample was measured at 405 nm wavelength.

### 2.7 Micellar aggregation characterization

The critical micelle concentration (CMC) of the antimicrobial lipid mixture was measured by characterizing the fluorescence emission properties of a hydrophobic probe molecule (1-pyrenecarboxaldehyde) in the presence of different mixture doses in PBS (pH 7.4) from Gibco (Thermo Fisher Scientific, Waltham, MA). Fluorescence spectroscopy measurements were performed using a SpectraMax iD5 microplate reader (Molecular Devices, San Jose, CA, USA), as previously described (Goddard et al., 1985). The CMC was determined by detecting changes in the probe's emission properties once micelles began to form in the solution. The measurement settings were probe excitation at 365 nm and emission monitoring in the range of 410 to 600 nm.

### 2.8 Membrane conductance experiments

Electrochemical impedance spectroscopy (EIS) measurements were conducted in PBS on tethered DOPC phospholipid bilayers that were formed on functionalized gold electrode surfaces, as previously described (Gooran et al., 2023). The experiments were performed using a tethaPod instrument (SDx Tethered Membranes, Sydney, Australia) with the following settings: 25 mV alternating current excitation, no offset voltage, and frequency sweep from 0.1 Hz to 2000 Hz. Time-resolved changes in the conductance signal were measured to track changes in membrane ionic permeability.

### 2.9 Statistical analysis

All tests were performed using the GraphPad Prism 10.2.2 software program (San Diego, CA, USA). One-way analysis of variance (ANOVA) with Dunnett's multiple comparisons test (*vs.* virus-only control for virological experiments and *vs.* lipid mixture at 0.5 × CMC for biophysical experiments) or linear regression analysis (dose-dependent effects) was used as appropriate. *P* < 0.05, *P* < 0.01, and *P* < 0.001 were defined as the levels of statistical significance (*, **, ***).

## 3 Results

### 3.1 Cell viability assessment

**Fig. 1:**
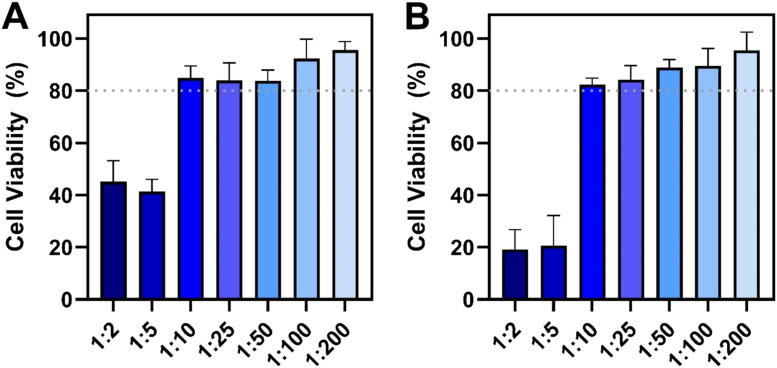
**Dose-dependent effect of antimicrobial lipid mixture on cell viability *in vitro*.** Effect of different mixture dilutions on the relative cell viability of **(A)** Vero and **(B)** MDCK cells as determined by MTT assay. Data are presented in terms of relative cell viability compared to mock-treated cells (no lipid) and reported as mean ± standard deviation from three independent experiments (*n* = 3 per group). Dotted lines indicate a 20 % drop in relative cell viability, which was defined as the cytotoxicity cutoff line (Freshney, 2015).

We first investigated the dose-dependent effects of the liquid-phase antimicrobial lipid mixture on the *in vitro* viability of Vero and MDCK cells, which were the cell lines used for viral infectivity experiments. In Vero cells, 1:2 and 1:5 dilutions of the mixture caused cell viability to drop by around 55–59 % and only 41–45 % viability was retained compared to mock-treated cells (Fig. 1A). On the other hand, the 1:10 and 1:25 dilutions maintained high cell viability levels around 84–85 %. The 1:50 and 1:100 dilutions also had high cell viability levels around 84 % and 92 %, respectively, while the cell viability was around 95 % upon treatment with the 1:200 dilution.

A similar trend in cell viability results was also observed with MDCK cells (Fig. 1B). As with Vero cells, the 1:2 and 1:5 dilutions caused appreciable cytotoxicity of MDCK cells and the resulting cell viability levels were around 20 % compared to mock-treated cells. Mixtures in the 1:10 to 1:50 dilution range maintained higher cell viability levels around 82–88 %. Upon treatment with the 1:100 and 1:200 dilutions, the cell viability levels were around 89 % and 95 %, respectively. These results led us to conclude that the 1:10 dilution and more dilute solutions were not cytotoxic and maintained acceptable cell viability levels (>80 %) to proceed with antiviral testing of the antimicrobial lipid mixture.

### 3.2 Antiviral activity in aqueous liquid conditions

**Fig. 2:**
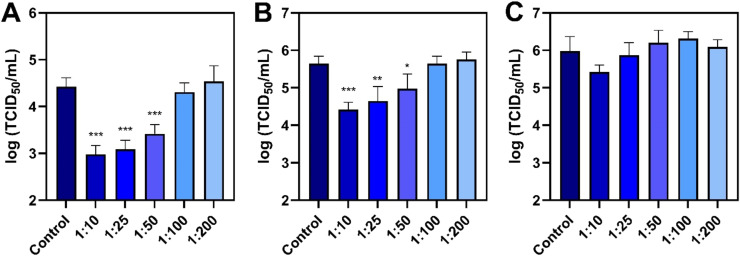
Virucidal evaluation of antimicrobial lipid mixture on enveloped and non-enveloped virus panel. Dose-dependent effect of antimicrobial lipid mixture on the infectivity of (A) enveloped ASFV, (B) enveloped IAV, and (C) non-enveloped EMCV viruses *in vitro*. Infectious viral titers were measured by CPE-based assay. Data are reported as mean ± standard deviation from three independent experiments (*n* = 3 per group). The markers *, **, and *** indicate *P* < 0.05, *P* < 0.01, and *P* < 0.001, respectively, *versus* the virus-only control.

We tested the virucidal properties of the antimicrobial lipid mixture against three viruses: enveloped ASFV and IAV and non-enveloped EMCV. First, we measured the dose-dependent effect of the antimicrobial lipid mixture on inhibiting ASFV infectivity in aqueous liquid conditions (Fig. 2**A**). In these virucidal experiments, ASFV and the lipid mixture were incubated together and then the ASFV-lipid mixture was diluted substantially before adding them to the cells. This virucidal testing protocol is focused on determining whether the direct interaction of ASFV with the lipid mixture affects viral infectivity and the lipid mixture was sufficiently diluted prior to the cell infection step so that it is inactive during subsequent steps of the viral infection cycle. For ASFV, the virus-only control had a mean titer of 4.42 log TCID_50_/mL and dose-dependent inhibition was observed. The 1:10 and 1:25 dilutions caused significant drops in the mean viral titer to around 2.98 and 3.09 log TCID_50_/mL, respectively, which correspond to >95 % reductions in viral infectivity. The 1:50 dilution was also virucidal and caused a decrease in the mean viral titer to 3.42 log TCID_50_/mL, which translates into a 90 % reduction in viral infectivity. By contrast, the 1:100 and 1:200 dilutions were inactive against ASFV and the resulting mean viral titers were around 4.31 and 4.53 log TCID_50_/mL, respectively, which are in the same range as the virus-only control.

Similar virucidal testing experiments were next conducted using IAV (Fig. 2B). The virus-only control had a mean viral titer of around 5.64 log TCID_50_/mL while the 1:10 and 1:25 dilutions caused titer reductions to around 4.42 and 4.65 log TCID_50_/mL, respectively. These results correspond to 94 % and 90 % drops in viral infectivity. In addition, the 1:50 dilution caused the mean viral titer to decrease to 4.98 log TCID_50_/mL, yielding a 78 % drop in viral infectivity. By contrast, the 1:100 and 1:200 dilutions were also not active against IAV and the mean viral titers were around 5.64 and 5.76 log TCID_50_/mL, which are similar to the virus-only control.

Together, these results support that the antimicrobial lipid mixture can inhibit ASFV and IAV, which are enveloped viruses. We also conducted similar experiments with non-enveloped EMCV and the mixture was judged to be inactive, with no tested dose causing a significant reduction in mean viral titer (Fig. 2C).

Motivated by these findings, we also conducted antiviral assay experiments to investigate whether the lipid mixture exhibits additional antiviral mechanisms besides virucidal activity (**Supplementary Fig. S1**). In these antiviral experiments, the ASFV suspension and the lipid mixture were incubated together and then the ASFV-lipid mixture was directly added to cells. Compared to the virucidal testing format described above, there was no dilution step prior to cell infection and hence the lipid mixture remained potentially active to inhibit ASFV during later stages of the viral infection cycle as well. According to this protocol, the virus-only control had a mean titer of 6.70 log TCID_50_/mL and dose-dependent inhibition by the lipid mixture was observed. The 1:10 dilution caused a significant drop in the mean viral titer to around 5.15 log TCID_50_/mL, which corresponds to ∼97 % reduction in viral infectivity and is nearly identical percentage-wise to the virucidal effect observed for this dose. The 1:25 and 1:50 dilution caused decreases in the mean viral titer to around 5.48 to 5.62 log TCID_50_/mL, which translate into ∼90 % reductions in viral infectivity. By contrast, the 1:100 and 1:200 dilutions were inactive against ASFV and the resulting mean viral titers were around 6.37 and 6.59 log TCID_50_/mL, respectively, which are similar to the titer of the virus-only control. These data support that the lipid mixture is mainly virucidal since the viral infectivity reductions in the virucidal and antiviral assay formats were similar. Notably, this finding is distinct from how solid-phase GML mitigant alone exhibits multiple mechanisms of antiviral activity, as indicated by previously reported larger infectivity drops in the antiviral assay compared to the virucidal assay (Jackman et al., 2020b). These mechanistic differences support that the lipid mixture has distinct antiviral properties compared to solid-phase GML mitigant.

**Fig. 3:**
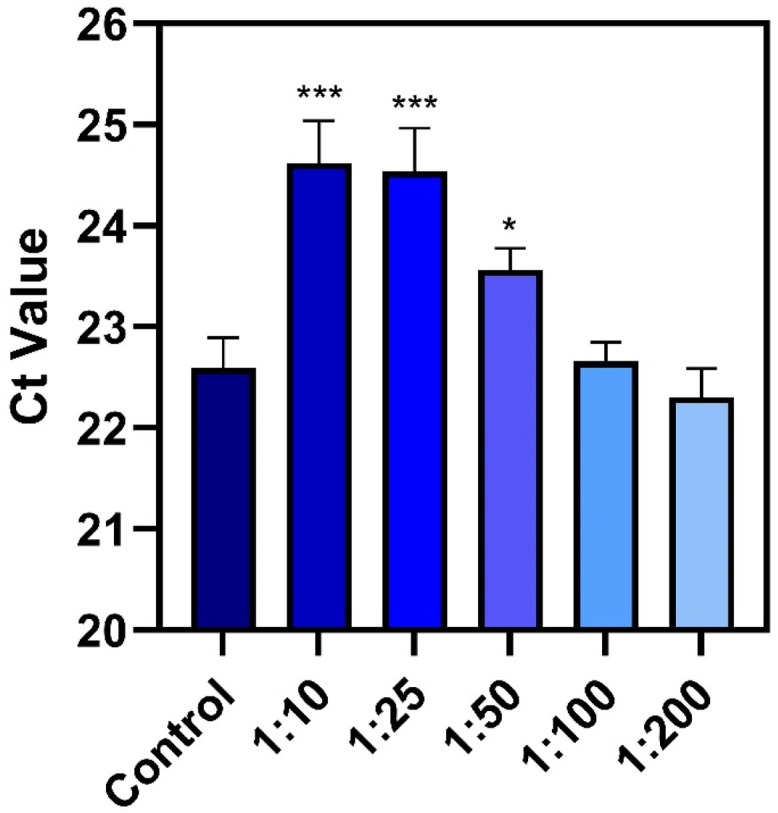
**PCR analysis of ASFV genetic material after virucidal treatment with antimicrobial lipid mixture.** Dose-dependent effect of antimicrobial lipid mixture on ASFV viral DNA *in vitro* according to the virucidal testing protocol. The amount of intact virus genetic material was determined by quantitative PCR assay. Data are reported as mean ± standard deviation from three independent experiments (*n* = 3 per group). The markers *, **, and *** indicate *P* < 0.05, *P* < 0.01, and *P* < 0.001, respectively, *versus* the virus-only control.

To further investigate the virucidal properties of the lipid mixture against ASFV in aqueous solution, we performed polymerase chain reaction (PCR) measurements to determine the relative amount of intact viral DNA extracted from treated ASFV suspensions (Fig. 3). Mechanistically, ASFV disruption involves envelope damage that affects viral infectivity and membrane-associated viral structural proteins while extensive loss of structural integrity could also release viral DNA, which would be more susceptible to environmental degradation upon release. Accordingly, in these experiments, a smaller Ct value indicates more intact viral DNA and *vice versa*. With a virucidal testing format, a Ct increase indicates a reduction in intact viral DNA due to the mixture treatment step.

The PCR measurements showed that the virus-only control had a Ct value of 22.6 while the 1:10 and 1:25 dilutions caused the Ct value to increase to around 24.6 and 24.5, respectively. Likewise, the 1:50 dilution caused the Ct value to increase to around 23.6 whereas the 1:100 and 1:200 dilutions had negligible effects, yielding Ct values around 22.3 to 22.6 that were comparable to the untreated sample. Together, these data support that the 1:10 to 1:50 dilutions reduced viral DNA levels based on the Ct value increase, which is aligned with the viral infectivity drops for those cases and reflect how lipid mixture-mediated ASFV disruption reduced intact viral DNA levels. Likewise, the 1:100 and 1:200 dilutions of the lipid mixture had no effect on viral DNA and those doses did not affect viral infectivity as well. These findings are noteworthy because they support that the virucidal lipid mixture impaired both viral infectivity and viral nucleic acids.

### 3.3 Antiviral activity in feed

These results led us to test whether the virucidal lipid mixture can be used as a feed additive to inhibit ASFV. The additive was mixed into feed at an inclusion rate of 0 wt%, 0.25 wt%, 0.5 wt%, 1 wt%, or 2 wt% and then the feed samples were spiked with ASFV for 30-min or 24-h incubation periods. Afterwards, the viral titer of ASFV recovered from the feed was measured.

**Fig. 4:**
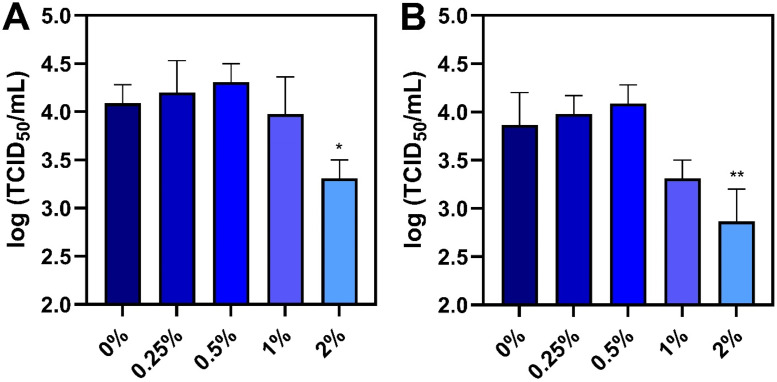
**Effect of antimicrobial lipid mixture on mitigating ASFV infectivity in feed**. Different inclusion rates of the lipid mixture were added to feed (0–2 wt%) and then ASFV was spiked into the feed for **(A)** 30 min or **(B)** 24 h incubation periods. Afterwards, infectious viral titers were measured by CPE-based assay. Data are reported as mean ± standard deviation from three independent experiments (*n* = 3 per group). The markers * and ** indicate *P* < 0.05 and *P* < 0.01, respectively, *versus* the virus-only control.

We first determined the viral titer recovered from ASFV-spiked feed samples after 30-min incubation (Fig. 4A). The mean titer of the virus-only feed sample without additive was around 4.09 log TCID_50_/mL while the feed sample containing 2 wt% additive had a significant decrease in the viral titer down to around 3.31 log TCID_50_/mL, which is equivalent to an 83 % drop in viral infectivity. By contrast, the feed samples containing 0.25 wt% to 1 wt% additive did not reduce viral infectivity.

We also measured the viral titer recovered from ASFV-spiked feed samples after 24-h incubation and a more pronounced antiviral effect was observed (Fig. 4B). The mean titer of the virus-only feed sample without additive was around 3.87 log TCID_50_/mL while the mean titer of the feed sample containing 2 wt% additive dropped significantly to around 2.87 log TCID_50_/mL, which corresponds to a 90 % drop and supports more extensive mitigation with longer incubation period as compared to the 30-min data. The mean viral titer of the feed sample containing 1 wt% additive also tended to decrease to around 3.31 log TCID_50_/mL and a dose-dependent trend in viral infectivity reduction was observed in this dose range (*P* < 0.01). By contrast, the feed samples containing 0.25 wt% to 0.5 wt% additive did not reduce viral infectivity levels. In summary, the 2 wt% additive dose had the greatest effects in terms of fast-acting within 30 min and reducing viral infectivity by the largest magnitude.

In addition, we investigated the effect of feed additive treatment on ASFV p72 antigen levels, which is a structural protein present on recovered ASFV particles (Liu et al., 2019) and whose conformational stability is affected by viral envelope disruption, as has been discussed for other types of enveloped viruses (Salimi et al., 2020). While antibodies can readily bind to structurally intact p72, conformational changes in the recognized p72 protein epitope can decrease antibody binding (Gomez-Puertas et al., 1996; Zsak et al., 1993). In ELISA experiments, a larger signal corresponds to more structurally intact p72 protein whereas a smaller signal indicates more disruption (Jackman et al., 2020b). Hence, these ELISA measurements provide insight into the extent to which the lipid mixture disrupts ASFV particles, which fits with past electron microscopy results on other enveloped virus types demonstrating that antimicrobial lipids such as GML in the mixture cause viral envelope disruption and loss of the virus particle's structural integrity (Thormar et al., 1987, 1994; Hierholzer et al., 1982).

**Fig. 5:**
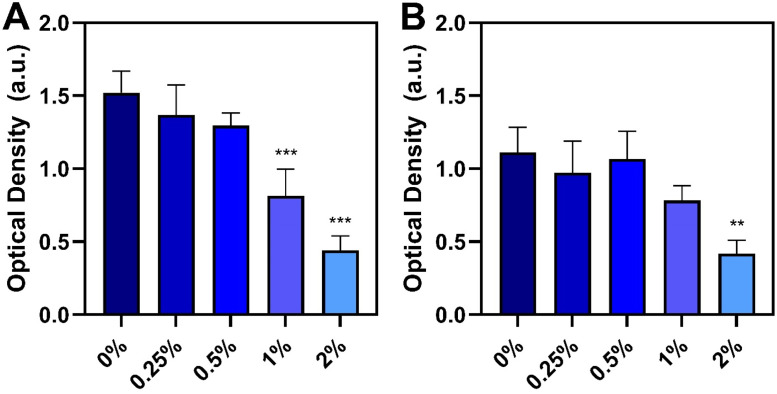
**Effect of antimicrobial lipid mixture on mitigating ASFV p72 antigen level in feed**. Different inclusion rates of the lipid mixture were added to feed (0–2 wt%) and then ASFV was spiked into the feed for **(A)** 30 min or **(B)** 24 h incubation periods. Afterwards, the relative amount of conformationally intact p72 protein antigen was measured by ELISA. The 0 wt% test group corresponds to the virus-only control. Data are reported as mean ± standard deviation from three independent experiments (*n* = 3 per group). The markers ** and *** indicate *P* < 0.01 and *P* < 0.001, respectively, *versus* the virus-only control.

We first measured p72 levels found in ASFV recovered from virus-spiked feed samples after 30-min incubation (Fig. 5A). The virus-only feed sample without additive had a mean OD value of 1.52 whereas 2 wt% additive caused a significant, markedly reduced OD value of 0.44 that corresponds to a 71 % reduction in conformationally intact p72. Notably, a dose-dependent decrease in signal intensity was observed across all tested doses (*P* < 0.001) and 1 wt% additive also caused a significantly reduced OD value of 0.81, which equates to 47 % reduction.

After 24-h incubation, the mean OD value of the virus-only feed sample without additive had decreased to 1.11 and the corresponding OD value of the virus-spiked feed sample containing 2 wt% additive was 0.42 (Fig. 5B). This significant difference translates into a 62 % reduction while the similar OD value magnitude after 30-min and 24-h incubation periods suggests that the maximum level of ASFV particle disruption occurred quickly. In terms of percentage changes, the mitigation effect after 24-h incubation appears smaller than the mitigation effect after 30-min incubation compared to their respective virus-only controls, but the absolute effect of viral mitigation is similar. The difference in relative mitigation effects is due to the duration of feed storage since longer incubation periods contribute to partial virus degradation even in the absence of mitigant (Dee et al., 2018). Inclusion of 1 wt% additive also tended to cause a marked decrease while 0.25 wt% and 0.5 wt% additive had negligible effect. Collectively, these findings support that the antimicrobial lipid mixture can be used as a feed additive to inhibit ASFV based on the measured decreases in viral infectivity and the conformational stability of the p72 protein.

### 3.4 Characterization of membrane-disruptive properties

To investigate the biophysical mechanism of antiviral activity, we also tested the membrane-disruptive properties of the antimicrobial lipid mixture. We first measured the critical micelle concentration (CMC) of the antimicrobial lipid mixture, which is the lowest mitigant dose at which membrane-disruptive micelles form. Below CMC, no micelles are present. The CMC was equivalent to a 1:8000 dilution, which led us to test the membrane-disruptive properties of the lipid mixture at 4 ×, 2 ×, and 0.5 × CMC concentrations that corresponded to 1:2000, 1:4000, and 1:16,000 dilutions, respectively.

**Fig. 6:**
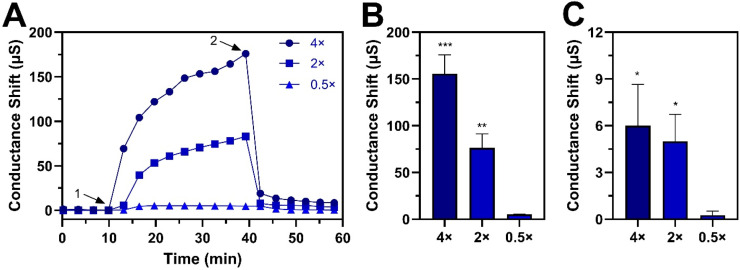
Biophysical characterization of membrane-disruptive properties of antimicrobial lipid mixture. Concentration-dependent effect of antimicrobial lipid mixture on membrane conductance of a tethered phospholipid bilayer platform based on (A) interaction kinetics, (B) maximum conductance shifts due to mixture treatment, and (C) final conductance shifts after buffer washing. For panel (A), different lipid mixture concentrations were added to the tethered phospholipid bilayer platform (arrow 1) and then washed away (arrow 2). For panels (B) and (C), data are reported as mean ± standard deviation from three independent experiments (*n* = 3 per group). The markers *, **, and *** indicate *P* < 0.05, *P* < 0.01, and *P* < 0.001, respectively, *versus* the 0.5 × CMC concentration set.

Accordingly, we conducted EIS measurements to determine the effects of antimicrobial lipid mixture treatment on the membrane conductance properties of a tethered phospholipid bilayer. When the lipid mixture at 4 × and 2 × CMC was added to the tethered bilayer, large increases in membrane conductance of 156 ± 20 and 76 ± 15 µS occurred, respectively (Fig. 6A,B). These results indicate extensive membrane permeabilization and dose-dependent effects.

After a buffer washing step, the resulting membrane conductance shifts were around 6.0 ± 2.6 and 5.0 ± 1.7 µS, respectively, which indicate major recovery of the membrane sealing while some degree of membrane disruption persisted (Fig. 6C). By contrast, when the lipid mixture at 0.5 × CMC was added to the tethered bilayer, there was an appreciably smaller increase in membrane conductance of around 5.3 ± 0.3 µS, which was fully reversible and returned to baseline values (0.3 ± 0.3 µS) after buffer washing. These data support that the antimicrobial lipid mixture disrupted phospholipid bilayers only above its CMC, which is consistent with the micelle-dependent antiviral mechanism of antimicrobial lipids (Jackman et al., 2023) and greater membrane disruption levels at higher micelle concentrations.

Of note, the lipid mixture caused mainly transient membrane disruption from the 1:4000 dilution upward whereas the mixture only exhibited virucidal activity from the 1:50 dilution upward. This difference in concentration ranges likely relates to two points. First, the effective membrane amount in the tethered bilayer assay is much smaller than the effective membrane amount of the virus particles in the antiviral assays so fewer micelles are needed to cause membrane disruption in the biophysical experiments. Second, the tethered bilayer results support that the membrane disruption caused by the lipid mixture at the tested concentrations is mainly transient (*i.e.*, return to near-baseline values upon buffer washing), which is likely insufficient to irreversibly damage virus particles to render them non-infectious. This finding is consistent with the virucidal behavior of the lipid mixture, which can not only reduce viral infectivity and affect membrane-associated viral structural proteins but also indirectly diminish viral DNA levels due to virus particle disruption, supporting a strongly disruptive mechanism that requires a relatively high mitigant concentration. From the biophysical experiments, we can conclude that the lipid mixture begins causing membrane disruption in a CMC-dependent manner while higher concentrations are needed for effectively mitigating enveloped viruses like ASFV.

## 4 Discussion

A key motivation driving the development of water-dispersible antimicrobial lipid mixtures is the growing number of studies that demonstrate orally delivered medium-chain antimicrobial lipids such as fatty acids and monoglycerides can inhibit animal viruses in *in vivo* disease prevention and treatment applications as described below. In those studies, a defined dose of lipid mitigant in a fixed volume was given via oral administration to animals and prior solubilization in organic solvent was a necessary step. The following application examples highlight these opportunities and challenges:

Yang et al. reported that orally administered monocaprylate (C_8_ monoglyceride) can reduce mortality and viral load as well as improve inflammatory responses among piglets infected with enveloped PRRSV *in vivo* (Yang et al., 2022). This result supports the merits of orally delivering medium-chain antimicrobial lipids while it was noted that the monocaprylate was first dissolved in dimethyl sulfoxide (DMSO) before dilution. Another study reported that orally administered GML had therapeutic effects to treat enveloped PEDV infection in piglets *in vivo* while the solubilization details were not described (Liu et al., 2022). In addition, it has been reported that GML could also therapeutically treat enveloped Seneca Valley virus (SVV) infection in piglets, and it was noted that DMSO was used to dissolve the mitigant (Su et al., 2023). Together, these findings demonstrate that orally delivered medium-chain antimicrobial lipids, especially monoglycerides, can be useful for animal health in the context of preventing and treating enveloped viral infections but water-dispersible formulations that do not require organic solvent solubilizers like DMSO are needed.

Such formulation capabilities would open the door to drinking water applications for lipid mitigants that can enable various application possibilities such as: (1) inhibiting pathogens in drinking water; (2) promoting animal health and overall wellbeing in terms of disease prevention along with microbiome management, immune function support, and aiding growth performance; (3) facilitating widespread delivery to livestock animals, *e.g.*, to curb disease spread in cases of potential outbreak; and (4) enabling targeted delivery to sick animals for disease treatment (Jackman et al., 2022; Page and Gautier, 2012; van der Wolf et al., 2017).

However, it has proven difficult to directly add these lipid mitigants to drinking water at the high concentrations needed for injector lines because they have relatively low solubility and high melting points. In this study, these considerations motivated us to develop a water-dispersible antimicrobial lipid formulation based on mixing medium-chain monoglycerides and lactylates with other natural, highly soluble molecules and did not require organic solvents. This liquid-phase mixture was stable in the stock solution, miscible with aqueous solutions, and readily dispersible across a wide dilution range, which is advantageous compared to a previously reported MCFA mixture (Tran et al., 2021) that contained C_8_ fatty acid (liquid-phase at room temperature by itself) and was active against ASFV as a feed mitigant but exhibited poor mixing in the stock solution and formed aggregates upon aqueous dilution when tested in comparison (see **Supplementary Fig. S1** and **Supplementary Table S1)**. Our findings further support that the GML-containing mixture can potently inhibit enveloped viruses like ASFV and IAV based on a membrane-disruptive mechanism. Importantly, the lipid mixture had excellent selectivity for inhibiting membrane-enveloped viruses and was nontoxic to mammalian cells in the antiviral dose range (at least 10-fold selectivity).

In the ASFV context, medium-chain antimicrobial lipid mitigants as feed additives have also demonstrated practical utility to stop virus transmission. For example, an MCFA feed additive has been reported to prevent ASFV transmission to pigs from virus-spiked feed and feed ingredients (Niederwerder et al., 2021). Motivated by these findings, in this study, we also verified that the mixture performed well at ASFV feed mitigation, demonstrating slightly better performance than solid-phase GML mitigant despite having a much lower effective inclusion rate of GML itself (Jackman et al., 2020b). The water-dispersible formulation used in this study establishes the feasibility of delivering medium-chain antimicrobial lipid mitigants in drinking water while further improvements in antiviral potency may be achieved by optimizing the mixture components (Moon et al., 2024; Yoon et al., 2020).

In closing, we also wish to briefly contextualize the utility of developing a water-dispersible antimicrobial lipid mixture compared to other mitigant options. Aqueous formulations of formaldehyde have long been used for feed pathogen mitigation and disinfecting contact surfaces but are not suitable for animal health applications (Dee et al., 2014; Le et al., 2022). Likewise, other commercial disinfectants, even when dispersible in aqueous solutions, are limited to treating contact surfaces and are not suitable for feed pathogen mitigation or animal health applications (Beato et al., 2022; Frost et al., 2023). On the other hand, a water-dispersible formulation of medium-chain antimicrobial lipids such as the one developed here is suitable for animal health applications, including oral delivery to animals, as well as for feed pathogen mitigation and contact surface disinfection.

## 5 Conclusions

These findings support that the water-dispersible antimicrobial lipid mixture has excellent potential for drinking water and feed applications related to enveloped virus disease prevention and mitigation, especially against ASFV. Future research possibilities include testing these antiviral activities in animal models of viral infection and disease transmission as well as refining the mixture composition to further increase the antiviral potency and potentially add more functionalities.

## Data availability

All relevant data are within the manuscript, figures, and supplementary data files.

## CRediT authorship contribution statement

**Joshua A. Jackman:** Writing – review & editing, Writing – original draft, Project administration, Funding acquisition, Data curation, Conceptualization. **Roza Izmailyan:** Writing – review & editing, Investigation. **Rafayela Grigoryan:** Writing – review & editing, Investigation. **Tun Naw Sut:** Writing – review & editing, Investigation. **Abel Taye:** Writing – review & editing, Investigation. **Hovakim Zakaryan:** Writing – review & editing, Project administration, Funding acquisition, Conceptualization. **Charles C. Elrod:** Writing – review & editing, Writing – original draft, Project administration, Conceptualization.

## Declaration of competing interest

The authors declare the following financial interests/personal relationships which may be considered as potential competing interests:

Charles C. Elrod is employed by and Joshua A. Jackman serves as a board member of the company Natural Biologics Inc., which develops natural feed additives for animal health applications. The other authors declare that they have no competing interests.

## Appendix Supplementary materials

Image, application 1
