# Supplementary material for: Development of a water-dispersible antimicrobial lipid mixture to inhibit African swine fever virus and other enveloped viruses
Source: Virus Res. 2024 Dec 25;351:199516. doi: 10.1016/j.virusres.2024.199516 (PMC11731633; doi:10.1016/j.virusres.2024.199516)
Supplement: Supplementary file 1 [file mmc1.docx]

**Supplementary Material**

**Development of a water-dispersible antimicrobial lipid mixture to inhibit African swine fever virus and other enveloped viruses**

Joshua A. Jackman,^a*^ Roza Izmailyan,^b^ Rafayela Grigoryan,^b^ Tun Naw Sut,^a^ Abel Taye,^a^ Hovakim Zakaryan,^b^ Charles C. Elrod^c*^

^a^ School of Chemical Engineering and Translational Nanobioscience Research Center, Sungkyunkwan University, Suwon 16419, Republic of Korea

^b^ Institute of Molecular Biology of NAS, Hasratyan 7, 0014, Yerevan, Armenia

^c^ Natural Biologics Inc., Newfield, NY 14867, USA

*Correspondence: jjackman@skku.edu (JAJ) and celrod@naturalbiologics.com (CCE)


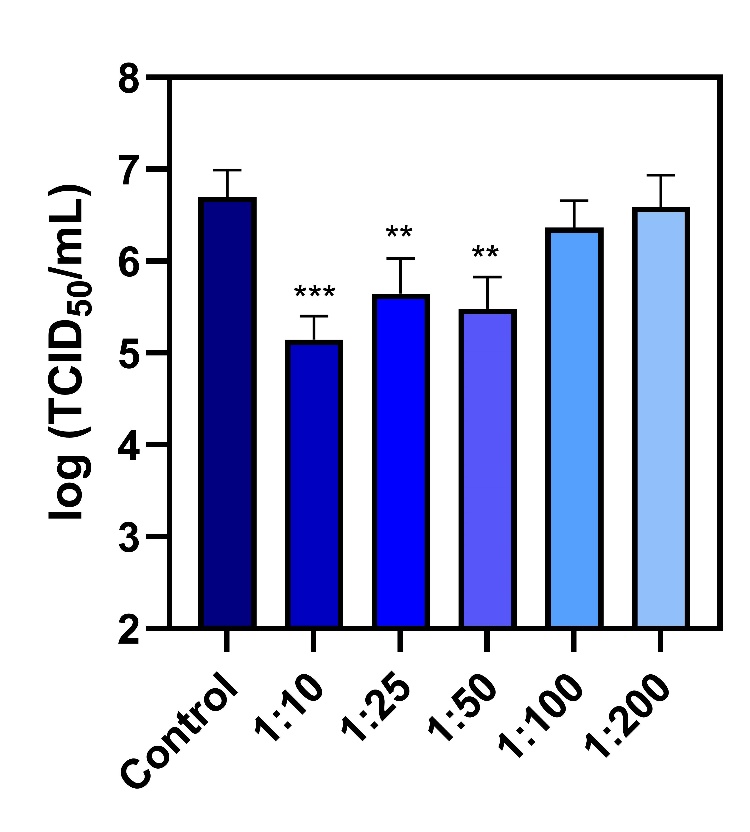


**Supplementary Figure S1.** **Antiviral evaluation of antimicrobial lipid mixture on ASFV infectivity.** Dose-dependent effect of antimicrobial lipid mixture on ASFV infectivity *in vitro*. Infectious viral titers were measured by CPE-based assay. Data are reported as mean ± standard deviation from three independent experiments (*n* = 3 per group). The markers *, **, and *** indicate *P*<0.05, *P*<0.01, and *P*<0.001, respectively, versus the virus-only control.

**
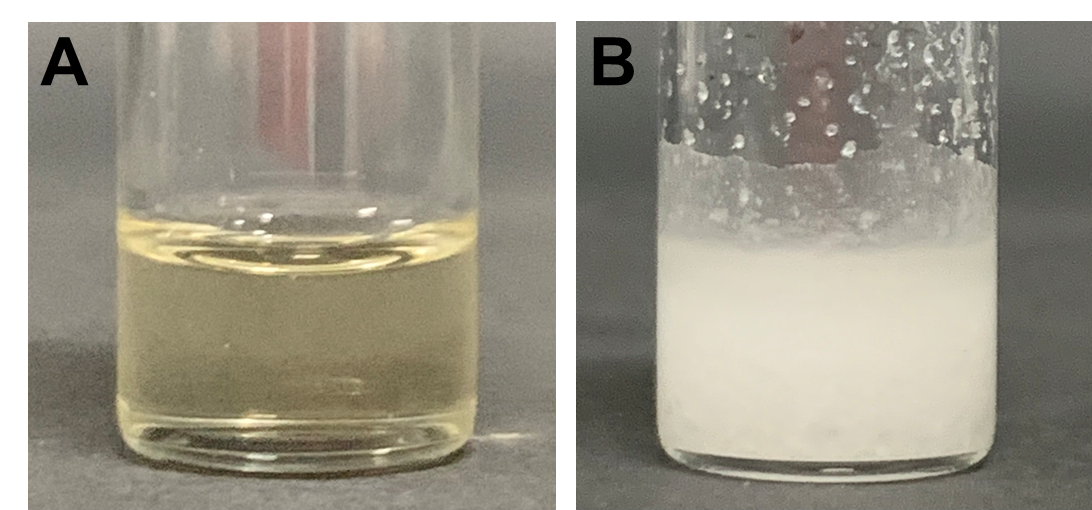
**

**Supplementary Figure S2. Photographs of antiviral mitigants at room temperature. (A)** Antimicrobial lipid mixture developed in this study, which is a clear and well-mixed liquid. There was high miscibility of molecular components. **(B)** Previously reported mixture of C_8_, C_10_, and C_12_ fatty acids based on a 1:1:1 mass ratio, which exhibits sticky character and cloudy aggregates with more gel-like properties. There was poor miscibility of molecular components. In these photographs, equal volumes of each mitigant sample were deposited in the respective vials.

| **Sample** | **Lipid mixture in this study** | **C_8_:C_10_:C_12_ fatty acids (1:1:1)** |
| --- | --- | --- |
| Stock | Soluble | Insoluble |
| 1:5 dilution | Soluble | Insoluble |
| 1:10 dilution | Soluble | Partially soluble (aggregates) |
| 1:15 dilution | Soluble | Partially soluble (aggregates) |
| 1:20 dilution | Soluble | Partially soluble (aggregates) |
| 1:25 dilution | Soluble | Partially soluble (aggregates) |
| 1:30 dilution | Soluble | Soluble |

**Supplementary Table S1. Comparison of mixing properties for different antiviral mitigants.** Data are reported based on visual observation of prepared samples after 10-min incubation at room temperature.
